# Dual role of inflammatory mediators in cancer

**DOI:** 10.3332/ecancer.2017.721

**Published:** 2017-02-23

**Authors:** TG Shrihari

**Affiliations:** Department of Oral Medicine and Radiology, Krishnadevaraya College of Dental Sciences and Hospital, Bengaluru-562157, Karnataka, India

**Keywords:** inflammation, cancer, myeloid derived suppressor cells, tumour associated macrophages, tumour microenvironment, tumour associated neutrophils, transforming growth factor- beta, vascular endothelial growth factor, micro RNAs, NF-kB, STAT3, AP-1, chemokines, growth factors, cytokines, tumour immunosurveillence, GM-CSF, matrix metallo proteinases

## Abstract

Inflammation is the body’s response to noxious stimuli such as infectious, physiological or chemical agents, it releases various inflammatory mediators via immune cells such as neutrophils, macrophages, and lymphocytes. These inflammatory mediators are growth factors, chemokines, and cytokines. Reactive oxygen species (ROS) and nitrogen species (RNS) activate transcriptional factors (NF-KB, STAT-3) and bring about cellular proliferation, genomic instability, angiogenesis, resistance to apoptosis, invasion, and metastasis. The presence of inflammatory mediators in the tumour microenvironment inhibits or promotes inflammation-induced cancer, depending on various stages of immune surveillance of the tumor i.e. by immunoediting, immunoprocessing, and immunoevasion. Myeloid derived suppressor cells are immature myeloid progenitor cells. They are the major immune-suppressor cells in the tumour inflammatory microenvironment that activate transcriptional factor NF-KB, STAT-3 to bring about tumour progression. Another gene which the micro RNA’s are noncoding RNA molecules is found to have a link with inflammation and cancer. This article discusses the roles of inflammatory mediators involved in antitumour or protumour activity within the context of the tumour microenvironment.

## Introduction

Inflammation is the body’s response to physical, chemical, thermal, or infectious stimuli and has a role in inhibiting or promoting cancer through immunosurveillance.

Cancer is a multifactorial process leading to structural and functional changes in a cell. Cellular changes include increased activity of oncogenes and loss of functional mutations in tumour suppressor genes can lead to cellular immortality, resistant to apoptosis, and carcinogenesis. The body’s immune defense mechanism plays a role in inhibition or promotion of cancer and its progression. In the extrinsic pathway of inflammation, inflammatory cells in the tumour microenvironment or the chronic inflammatory process because of physiological, infectious or chemical agents can lead to the progression of cancer. About 25% of cancers are associated with chronic inflammation or infectious agents, 30% of cancers can be associated with tobacco smoking and inhaled pollutants (such as asbestos and silica),and 35% of cancers can be associated with dietary factors. Some of the chronic inflammatory conditions leading to cancer are H Pylori infection leading to gastric carcinoma; Hepatitis B or C associated hepatocellular carcinoma, oral squamous cell carcinoma induced by lichen planus, gingivitis, or chronic periodontitis, salivary gland carcinoma-induced by sialadenitis. Most malignancies are associated with the inflammatory microenvironment converted into the protumourigenic microenvironment. PAMPs or DAMPS expressed on pathogens or tissue injury are recognized by a specialized pattern expressed on the host known as Pattern recognition receptors (PRR), including NOD-like receptors, TLR, trigger receptors on myeloid cells, inflammation-induced activation of innate immune cells and drive inflammation-induced cancer by production of cytokines mediated NF-kB activation [1–3].

In the intrinsic pathway of inflammation-induced cancer, MYC and RAS family oncogenes remodel the tumour microenvironment by activating transcriptional factors through recruitment of leucocytes, lymphocytes, chemokines, cytokines expression, and angiogenic switch induction. Oncoproteins such as (MYC,RAS ,RET) drive pro-inflammatory cytokines and chemokines (1L-8, 1L-6, 1L-1 Beta, CCL2,CCL20) by activation of signalling pathway.

Neutrophils are the first cell to migrate in the early stage of inflammation regulated by macrophages and mast cells in the tissues. Various types of leucocytes, the majority of them macrophages and lymphocytes, are activated and recruited to the inflammatory site at a later stage of inflammation by a signalling network involving cytokines, chemokines, and growth factors for defensive action against infection [1, 3–5].

## Inflammatory mediators which prevent the progression of cancer in the inflammatory milieu

**Figure 1.**
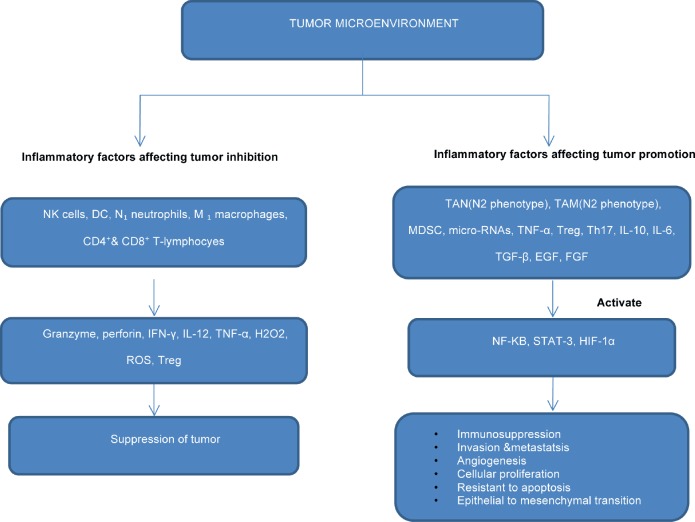
Flowchart showing the role of inflammatory mediators in inhibition or progression of cancerthrough activation of transcriptional factors.

N1 phenotypic neutrophils are classically activated and possess antitumour activity (Figure 1). It is recruited to the tumour microenvironment by chemokines such as CXCL8,CXCL1,CXCL2, and CXCL3 by the production of inflammatory mediators such as TNF-Alfa, MIP-1 ALFA, H202, and NO that are cytotoxic to tumour cells.

Another inflammatory mediator Pleiotropic cytokine interleukin -1 (IL-1) produced by tumour associated macrophages and mast cells, a group of 11 cytokines, has an antitumor activity because of its ability to activate and target innate and adaptive immune cells, which can inhibit tumour progression.

IL-10 produced by all immune cells, including monocytes, macrophages, mast cells, granulocytes, T and B lymphocytes, dendritic cells, keratinocytes and tumour cells induces anti-inflammatory or antitumor activity by enhancing B-cell proliferation, antibody production and survival, NF-kb inhibition, and also inhibiting production of TNF-alfa,IL-6,IL-12. IL-10 production from CD25 Tregs, regulates chronic infection and immune response, thereby preventing inflammation-induced cancer [6–8].

M1 phenotype macrophage classically activated macrophages release CXCL9 and CXCL10 chemokines and recruit Th1 lymphocytes which secrete IL-2,IL-12. TNF-Alfa, having IL-12^hi^ and IL-10^low^ phenotype, induces antitumour activity by increasing IL-12 production and decreasing IL-10 production on activation by LPS and INF-Gama [4, 7].

High production of free radicals, such as ROs, RNs during high oxidative stress by TNF-Alfa, TAMs, TAN causes inhibition of NF-kB expression, H2O2 formed during oxidative stress degrades the subunit 1kB -alfa thereby inhibiting inflammation and progression to cancer.

CD4 T cells express surface marker CD25 and FOXP3 transcriptional factors termed as Tregs, recruited by CCR4 and CCR7 chemokines, secrets cytokines IL-10 and TGF- Beta, involved in immunosuppression and inhibit inflammation in the tumour microenvironment leading to delay or prevention of inflammation-induced cancer [8–13].

Natural killer cells have antitumour activity by producing granzyme and perforin, in turn expressing NKG2D receptor on tumour and NK cells, which activate NK cells. These are the only natural immune cells which do not have protumourigenic activity [2].

Dendritic cells are antigen presenting cells, and they present antigen for activation of CD4+ T cells and CD8+ T cells [14].

Natural killer T cells divided into Type1 Natural Killer T cells have antitumour activity [13].

TGF-BETA have an anti-inflammatory activity and tumour suppressor effect through up-regulation of cyclin-dependent kinase inhibition (CKI) P21 and C-MYC Proto-oncogene down regulation [11, 15–17].

The CoX-2 enzyme,which produces a bioactive lipid prostaglandin E2, belongs to the prostanoid lipid family. This is a subclass of eicosanoids produced by the tumour and myeloid cells. COX-2 is expressed on all types of tumour cells. It is induced by mitogenic or pro-inflammatory stimuli, including growth factors and cytokines such as IL-1 Beta, IL-6, or TNF-Alfa, therefore overexpressed during inflammation. PGE2 promotes the activity of Th17 cells, and recruits neutrophils and monocytes to the site of inflammation. It contributes to resolution of acute inflammation. PGE2 has antitumour immunity by MDSC and Treg cell activation and expansion thereby inhibiting inflammation-induced cancer [15, 18–21].

IL-17 cytokine antitumour activity depends on the production of IFN-Gama by CD8+ T cells, recruited by chemokine CCR6. CD4 T cells preferentially differentiate into the Th17 T cell subset by IL-6 and TGF-Beta cytokine signals mediated by STAT-3 activation. IL-17 is activated by cytokine IL-23 which is the key factor for expanding and maintaining Th17 inflammatory population. IL-23 is closely associated with IL-12, and shares subunit IL-12 P40 with IL-23 that is involved in IFN-Gama production by Th1 and antitumour immune activity. It also possess the property of plasticity and shares the feature of Tregs by expressing CTLA-4, CD25, and FOxP3, limiting inflammation and immunosuppressive action [15, 22–24].

## Inflammatory mediators which promote the progression of cancer in the inflammatory milieu

Tissue repair from antimicrobial tissue damage by inflammatory mediators PGE2, ROS, TGF-Beta, and nitrogen intermediates has a dual role in aggravating and suppressing inflammation. Macrophages, dendritic cells, and phagocytes responsible for resolution of inflammation by the process of phagocytosis and apoptosis promote an anti-inflammatory response. If inflammation or the inflammatory process is aggravated, dysregulated chronic smouldering inflammatory cellular response release various inflammatory mediators such as growth factors, cytokines, reactive oxygen and nitrogen species from inflammatory cells such as neutrophils, macrophages, mast cells, MDSC, T and B lymphocytes leads to cell proliferation, angiogenesis, genomic instability, cell survival, immunosuppression, invasion and metastasis by activation of transcriptional factors such as NF-kB, STAT-3 and HIF-1 Alfa [9–11, 25].

Immune cells have an important role in inhibiting or promoting cancer through immune surveillance of tumours by the mechanism of immunoediting, immunoprocessing, and immunoevasion. Immunoevasion is one of the hallmarks associated for a tumour progression. Mechanisms involved in immunoevasion are the production of immunosuppressive cytokines, T cell apoptosis or loss of HLA class 1, and costimulatory molecules. In the immunoediting stage, high immunogenicity tumours eliminate tumours by NK cells, macrophages, dendritic cells, and T lymphocytes. Macrophages are the main abundant phagocytic cell lineage of the immunity found in the tumour microenvironment involved in antitumour immunity. Natural killer cells have the ability to recognise and lyse tumour cells by two mechanisms, i.e. either by perforin and granzyme release. Apoptosis induction by TNF ligands and IFN-gamma release inhibit tumour cell proliferation [19, 26].

Low level production of free radicals such as ROS and RNS during oxidative stress, in inflammatory conditions, activates the NF-kB signalling pathway which induces carcinogenesis by angiogenesis, cell proliferation, resistant to apoptosis, invasion, and metastasis [12, 17].

Dendritic cells (DCs) are antigen-presenting cells which rapidly respond to microenvironment signals. They turn into mature antigen capture dendritic cells and cross-priming to T and B lymphocytes to generate immunological memory. They have an essential role in antitumour adaptive immune responses [3, 13].

On activation, CD8 cytotoxic T cells (CTLs) eliminate tumour cells directly by the production of IFN-Gama, whereas CD4 T helper cells stimulate B cells supporting cytotoxic and humoral immune response [14].

Reduced tumour cell variant immunogenicity favours tumour progression by immunosuppression or resistance to immune attack. The immunoprocessing stage, genetic instability, and heterogeneity of cancer cells favour promotion of the tumour which is poorly recognised by the immune system or immunosuppression. In the immunoescape stage, by expression of MHC 11 and costimulatory molecules, there is antigen processing dysregulation. This leads to low level expression of tumour antigens and enhances other mechanisms of immunosuppression by development of T -cell tolerance to the tumour antigen and immunosuppressive cytokines such as IL-10, TGF-Beta or Tregs (Regulatory T cells) [9–11]. Treg cells are derived from T-cells mediated by TGF-Beta from tumour cells, which increases the growth and proliferation of Treg cells. It plays an important role in immunosuppression, self-tolerance, and immune homeostasis to different allergies, infectious diseases and cancer. Treg prevents NK cells,CD4+ and CD8+ T cells in order to promote tumourigenesis, and it also has an antitumour activity by suppressing inflammation. Mast cells present in the tumour microenvironment secrete IL-17 proinflammatory cytokine, enhance NF-KB and AP-1 activity, inhibit NK and T cells, and promote angiogenesis by the production of vascular endothelial growth factor (VEGF). IL-17 and IL-22 cytokines generated by CD4+ Th17 cells produce inflammatory mediators such as TNF-Alfa, IL-6 and IL-1 beta pro-inflammatory cytokines and promote tumourigenesis by production of PGE2,VEGF, keratinocyte-derived chemokine, and macrophage inflammatory protein -2 (MIP-2) angiogenic factors. IL-17 and IL-22 enhances IL-23 protumourigenic activity which enhance tumour growth and survival through JAK/STAT3 by activation of IL-6 results in cellular proliferation, angiogenesis, and tumour progression. IL-17 also acts as an antitumour immunity through the production of cytokines and promoting the activity of CD4+ and CD8+ T cells and immune response.

IL-1 Beta in the tumour site is produced by tumour and immune cells involved in the generation of MDSC and their migration to the site of the tumour, and it induces COX-2 expression which prevents maturation and activation of antigen presenting cells at the tumour site involved in the upregulation of TNF-Alfa, MDSC immunosuppression, carcinogenesis, tumour growth, invasion and metastasis in chemically-induced tumours. [3, 5, 19].

Tumour-associated neutrophils (N1 phenotype) exhibit antitumour activity in the early phase of the tumour through T cell responses by inhibiting TGF-Beta signalling,production of TNF-Alfa, NO, and H2O2 recruited to the tumour by chemokines such as (CXCL8, CXCL1, CXCL2, CXCL3, and CXCL5) and protumoural activity by TGF-Beta (N2phenotype) through the release of neutrophil elastase. ROS, and RNS promote genomic instability and mediate angiogenesis by release of VEGF, CCL17 recruiting Treg in the tumour, MMP-9, or Prokinectin -2, and neoplastic cell invasion through the release of soluble factors (Eg-HGF and Oncostatin). The tumour-associated neutrophil phenotype (N1-N2) is influenced by the evolution of the tumour microenvironment. The existence and properties of the N1 or N2 phenotype in cancer-associated inflammation in humans need to be studied [5, 14, 26].

Mononuclear phagocytes have a property of plasticity in response to the physiological and functional demand of macrophages polarised activation. Microbial stimuli (LPS), cytokines (TNF- Alfa and GM-CSF) or Interferon Gamma mediated activation of M1 classically activated macrophages that have an antitumour activity by high production of IL-12,IL-23, and low production of IL-10. They also activate tissue destructive properties by releasing chemokines such as CXCL9 and CXCL10 which attract Th1 lymphocyte. Alternative activated M2 macrophages (tumour associated macrophages) activates Th2 phenotypic lymphocytes. Induced by cytokines IL-4, IL-10, IL-13 down regulating IL-12 and MHC-Class 11 and release CCL17 ,CCL22 and CCL24 chemokines, which recruit Treg cells, basophils, eosinophils, B lymphocytes, bring about immunoregulation by suppressing Th1 response and poor antigen presentation, and also promote tumour progression by producing V EGF, IL-10 and TGF-Beta [3, 13, 26].

Tumour-associated macrophages (TAM) have a TAM1 phenotype in the early stages of the tumour and promote tumour progression by secreting proinflammatory cytokines such as IL-6, TNF-Alfa,IL-23, and iNOS, involved in tumour initiation and tumour promotion. TAM are phenotypically and functional similar to M2 macrophages. M2 polarisation induced by chronic inflammatory mediators IL-10,TGF-Beta, PGE2 and CSF-1, promotes tumour progression by releasing various mediators such as cytokines (IL-10,TGF-Beta, TNF-Alfa, IFN-Beta, PGE2,ROS), chemokines ( CCL18, CCL22, CCL17, CXCL8), growth factors (VEGF, PDGF, EGF, FGF, M-CSF) and proteinases (MMPs, Cathepsins) [15, 19, 22, 23, 25].

PGE2 is involved in cellular proliferation by the activation of Ras–MAPk, NF-kB,P38, STAT-3 signalling cascade, DNA mutation by ROS, angiogenesis by enhancing VEGF-A, immunosuppression by Tregs and MDSC activation and expansion, apoptosis resistance by Bcl-2 and Bcl-XL, and metastasis by NF-kB-mediated MMP activation [21].

In the advanced stage of cancer, TGF-Beta mutational inactivation results in immunosuppression, epithelial to mesenchymal transition induced invasion and metastasis mediated by SMAD, Slug, Snail, transcription factors and MAPKs pathway.

IL-10 mediated pro-tumoural activity by transcriptional factor STAT-3 activation results in BCL-2 or BCL-XL anti-apoptotic gene up-regulation, cell proliferation by cyclinD1, D2, B and proto-oncogene C-MYC. Immunosuppressive activity on macrophages and dendritic cells, dampen antigen presentation, cell maturation and differentiation results in tumour immune evasion.

B cells do not infiltrate precancerous tissue, cytokines, or antibodies produced by lymphocytes induced cancer-promoting inflammation. B-lymphocytes in the tumour microenvironment promote tumour progression by myeloid and mast cell activation and proinflammatory cytokine production such as lymphotoxin. Immunosuppressive plasmocytes (ISPCs) suppress cytotoxic T lymphocytic activation by the production of IgA, PDL-1, and IL-10 [5, 13].

Natural killer type 2 cells have protumourigenic activity by the production of IL-13 leading to MDSC accumulation [13].

MDSC are a heterogeneous population of immature myeloid cells, consisting of precursors for granulocytes, macrophages, or dendritic cells, derived from myeloid progenitor cells of bone marrow, involved in chronic inflammation and tumour progression. GMCSF (Granulocyte- macrophage colony stimulating factor), IL-10,IL-6,VEGF, TGF-Beta, COX-2, and HIF-1Alfa are key factors in the generation and expansion of MDSC. CCL2, CCL3, CCL4, CCL5, CXCL1, CXCL8 etc. are chemokines involved in the recruitment of MDSC to tumour microenvironment. It reduces T cell activation and function by Arginase -1, iNOS, peroxynitrate overexpression and cysteine depletion. Myeloid-derived suppressor cells secrete IL-10, IL-6, TNF-Alfa, IL-1 beta, COX-2, IFN-Gama,HIF-1 Alfa and ROS, skew the CD4+ T Cells to Tregs and suppress the antitumour immunity of CD4+, CD8+, and NK cells, resulting in the suppressive and tolerogenic environment and M2 phenotypic polarisation of tumour-associated macrophages favouring tumour progression. MDSC mediated cellular proliferation, angiogenesis by activation of STAT-3, invasion by MMP through degradation of the extracellular matrix or basement membrane and epithelial to mesenchymal transition by secretion of TGF-Beta, HGF, and FGF [16, 17, 27, 28].

Tumour-associated macrophages reduce antimicrobial and antitumour activity by expressing low levels of MHA Class11, increase angiogenic mediators such as VEGF and COX2 derived PGE2 and IL-10 anti-inflammatory cytokine. It is also noted that on the other side IL-10 also acts as antitumour activity by suppressing inflammation. Low expression of IL-12 by alternatively activated macrophages (M2 type) expressed by tumour associated macrophages is the hallmark feature. The master transcriptional regulators of tumour-associated macrophages are NF-KB and HIF-1(Hypoxia-inducible factor-1) which are central regulators of tumour progression and metastasis by matrix metalloproteinases. NF-KB activation in cancer cells by IL-1, TNF-Alfa proinflammatory cytokines, hypoxia, and ROI is expressed by leukocytes as well as the pathogen-associated molecular pattern (PAMP) and STAT-3 transcriptional factor, activated by IL-6 and EGF which in turn up-regulates HIF-1 responsible for cancer cell survival and tumour progression by cell proliferation, angiogenesis, genomic instability through anti-apoptotic activity, epithelial-mesenchymal transition, invasion, and metastasis [15, 20, 22, 29].

NF-kB and STAT-3 transcriptional factors interfere with synthesis of P53 and attenuate genomic surveillance mediated by P53, promoting tumour progression.

ROS and RNS produced from macrophages and neutrophils or released intracellularly by inflammatory cytokines IL-4, IL-1 Beta,TNF,IL-13 and TGF-Beta are shown to induce ectopic expression of activation-induced cystidine deaminase, a member of the DNA and RNA cytosine deaminase family, which has a crucial role in the mutation of TP53, MYC, and promotion of oncogenesis [18, 27].

Cellular susceptibility to mutagenesis may be increased directly by inflammation and indirectly by increasing the genomic destabilisation by disruption of cell cycle checkpoint and DNA repair pathway downregulation, which leads to genetic alteration [12].

Mismatch repair (MMR) proteins prevent microsatellite instability (MI) which induces genomic instability. Downregulation of MMR expression genes, MSH2, and MSH6 by inflammatory mediators such as ROS, PGE2, TNF, HIF-1 Alfa, IL-1 Beta results in accelerating DNA replication rate errors throughout the genome. MMR proteins activity is reduced by ROS, MMR protein MLH1 and tumour suppressor protein P53. Silencing of P53 in the inflammatory microenvironment, induced by proinflammatory cytokine such as 1L-6 and NO, and increased enzyme activity of DNA-hypermythylase promote hypermythylation. P53 inactivation mediated genomic surveillance induced by inflammation in turn, induces release of COX2 , NO produced by NOS2, and overexpression of MYC and BCL-2,results in the suppression of the DNA repair mechanism, and increases the cancer cell mutation rate [2, 30].

MicroRNA’s (miRs) are noncoding RNA molecules. They are posttranscriptional regulators as mir-155, mir196, mir210, mir21, and mir125b and participate in innate and adaptive immune response. They are responsible for activation and silencing of genes found in all biological fluids such as blood, urine, and breast milk. They have a role in inflammation-induced cancer by activation of transcriptional factors NF-kB, Activator protein 1 (Ap-1), and STAT-3, which promote cell proliferation, cell survival, angiogenesis, oncogene addiction, invasion, and metastasis [24, 31, 32].

Chronic inflammatory mediators released from immune cells such as macrophages, lymphocytes, and MDSC are cytokines (IL-6,IL-4,IL-8 and IL-10), growth factors (EGF,FGF,TGF-Beta), transcriptional factors (NF-KB, STAT-3 and HIF-1 ALFA), proteolytic enzymes (MMP-2,9,UPA) and COX-2 are all which drives the genomic instability, cellular proliferation, angiogenesis, resistance to apoptosis, epithelial to mesenchymal transition, invasion, and metastasis [33]. Identification of inflammatory mediators which promote or prevent the progression of cancer depends on the context of the tumour microenvironment. In the future, identification of inflammatory cancer biomarkers for early detection of cancer and prognostic purpose would be helpful for designing therapeutic drugs.

## Conclusion

Inflammation has a dual role in antitumour or protumoural activity depending on the plasticity of immune cells , their phenotype in the inflammatory tumour microenvironment, and their secreting factors such as cytokines, chemokines, growth factors and proteolytic enzymes. Inflammatory mediators such as cytokines, chemokines,growth factors, proteolytic enzymes produced by immune cells such as macrophages, neutrophils, and lymphocytes activate transcriptional factors and further downstream signalling pathway within the context of the tumour microenvironment. This later brings about tumour progression. Identification of inflammatory mediators and their interactions with other tumours and stromal cells within the tumour microenvironment involved in antitumour or protumour activity is important for future therapeutic strategy and tumour prognostic purpose.

## Abbreviations

HGF, Hepatic growth factor, VEGF, Vascular endothelial growth factor, MMP-9, Matrix mettaloproteinases-9, COX2, Cyclo-oxygenase2, INOS, Inducible nitric oxide synthase, ROS, Reactive oxygen species, PDGF, Platelet derived growth factor, EGF, Epidermal growth factor, FGF, Fibroblast growth factor, TNF-Alfa, Tumour necrosis factor-Alfa, IFN-Beta, Interferon Beta, IL-10, Interleukin 10, TGF-Beta, Transforming growth factor- Beta,CCL17,CC Chemokine ligand 17, CCL18,CC Chemokine ligand 18, CCL22, CC chemokine ligand 22, PGE2, Prostaglandin E2, IDO, Indoleamine 2,3 –dioxygenase, IL-2,Interleukin 2, IL-4, Interleukin 4, IL-6,Interleukin -6, IFN-Gamma, Interferon Gamma, COX-1, Cyclo-oxygenase 1, COX2, Cyclo-oxygenase 2, NF-KB, Neuclear factor KB, MCP-1, Macrophage/Monocyte chemoattractantprotein-1, M-CSF, Macrophage colony stimulating factor, IL-17, Interleukin 17, CD4+ Th17, CD4+ T helper lymphocyte17,MDSC, Myeloid derived suppressor cells,GM-CSF, Granulocyte Macrophage- Colony stimulating factor, G-CSF, Granulocyte colony stimulating factor, STAT3,Signal transducer and activator of transcription 3, bFGF- basic fibroblast growth factor, MMPS, Matrix metallo proteinases, HIF-1 Alfa, Hypoxia- Inducible factor Alfa, T reg cell, Regulatory T cell ,T h1, T helper1, Th2,T helper 2,TAM, Tumour associated macrophages, microRNA’s, miRs, MMR, mismatch repair, MI, microsatellite instability, HLA, human leucocyte antigen, MHC 1, major histocompatibility antigen 1.
